# The factor structure of the Barratt Impulsiveness Scale (BIS-11) and correlates of impulsivity among outpatients with schizophrenia and other psychotic disorders in Singapore

**DOI:** 10.1186/s12888-022-03870-x

**Published:** 2022-03-31

**Authors:** Jue Hua Lau, Anitha Jeyagurunathan, Saleha Shafie, Sherilyn Chang, Ellaisha Samari, Laxman Cetty, Swapna Verma, Charmaine Tang, Mythily Subramaniam

**Affiliations:** 1 Research Division, Institute of Mental Health, Buangkok Green Medical Park, 10 Buangkok View, Singapore, 539747 Singapore; 2 Medical Board, Institute of Mental Health, Buangkok Green Medical Park, 10 Buangkok View, Singapore, 539747 Singapore; 3 Department of Early Psychosis Intervention, Institute of Mental Health Buangkok Green Medical Park, 10 Buangkok View, Singapore, 539747 Singapore

**Keywords:** Impulsiveness, BIS-11, Schizophrenia, Outpatients, Singapore

## Abstract

**Background:**

Impulsivity has been linked to risky behaviours amongst patients with schizophrenia or other psychotic disorders. However, there is a dearth of studies examining impulsivity amongst this population in Singapore. Moreover, to date, scales to measure impulsivity have not been validated in this population. The present study seeks to examine the underlying factor structure of the Barratt Impulsiveness Scale (BIS-11) and explore sociodemographic and clinical correlates of impulsivity within this group.

**Methods:**

Confirmatory factor analyses (CFA) were conducted to test factor structures of the BIS-11 proposed in extant literature. However, due to poor fit statistics, the sample (*n* = 397) was split into two groups, with Exploratory Factor Analyses (EFA) conducted in the first subgroup (*n* = 200). The final model of the EFA was then tested within the second subgroup (*n* = 197) with CFA. Multivariable linear regressions were conducted to examine sociodemographic and clinical correlates of each underlying factor.

**Results:**

CFA indicated a three-factor structure amongst 16-items of the BIS-11 with acceptable fit: i) Non-planning impulsivity (5-items; α = 0.94), ii) Motor impulsiveness (6-items α = 0.84), and iii) Lack of self-control (5-items, α = 0.85). Lower education was associated with higher non-planning impulsivity. While age, ethnicity, marital status, and general psychiatric symptom severity were significant correlates of motor impulsiveness, problematic alcohol use and general psychiatric symptom severity were related to a greater lack of self-control.

**Conclusion:**

Factor structures of the BIS-11 suggested by extant literature were not applicable, and we propose an alternative factor structure for BIS-11. Significant correlates of impulsivity are highlighted, and avenues for future research are suggested.

**Supplementary Information:**

The online version contains supplementary material available at 10.1186/s12888-022-03870-x.

## Background

Impulsivity is a multidimensional construct defined as ‘a predisposition toward rapid unplanned reactions to internal or external stimuli without regard to the negative consequences of these reactions to themselves or others’ [1]. Researchers have long considered excessive impulsivity to be a core feature of schizophrenia and psychotic disorders. For example, several studies utilizing self-report measures have found that individuals with schizophrenia or other psychotic disorders were more impulsive than healthy controls [2, 3]. Moreover, Iancu et al. revealed that the general psychopathology and positive symptoms of schizophrenia were positively correlated with impulsivity [4]. Neurocognitive studies have also demonstrated impairments in frontal/executive brain regions associated with inhibitory and response inhibition processes related to impulsivity amongst these patients [2, 5]. Similarly, behavioural studies revealed that patients with schizophrenia perform poorly on impulsivity measures such as the stop-signal task [6, 7] or delay discounting measures [8].

Impulsivity has generally been viewed as counterproductive to society and linked to socially deviant behaviours such as aggression [9], substance abuse/dependence [10], and alcoholism [11] within the general population. Studies indicate that individuals with higher impulsivity are more likely to engage in risk-taking behaviour because they lack the self-control to refrain from doing so [12]. Among those with schizophrenia, higher impulsivity has been associated with increased risk of violent, aggressive, and/or antisocial behaviours [13, 14]. Individuals with schizophrenia who had lifetime alcohol abuse/dependence were found to have higher impulsivity and sensation-seeking than those without comorbid alcohol abuse/dependence [15]. In addition, schizophrenia has also been associated with an increased risk of violent behaviors compared to other psychiatric illnesses [16, 17] and the general population [18]. Given how impulsivity is associated with risky or harmful behaviours, this construct is particularly important to mental health researchers. Understanding the relationship between impulsivity and high-risk behaviours may allow for the adaptation or development of preventive or therapeutic strategies to ameliorate the quality of life for individuals with schizophrenia and reduce risk-taking behaviours.

However, before understanding the relationship, measures of impulsivity must be validated within this population. One widely used measure is the Barratt Impulsiveness Scale (BIS-11) [19]. A study by Zhornitsky et al. found that impulsivity was significantly higher in patients with schizophrenia, substance use disorder, and those with a dual diagnosis compared to healthy controls using the BIS -11 [20]. Another study conducted by Nolan et al. similarly found that patient**s** with schizophrenia scored higher on impulsivity than healthy controls [7]. However, psychometric studies have suggested that the original factor structure of the BIS-11 may not be valid and have proposed alternatives [21–23]. A systematic review by Vasconcelos et al. suggested that the number and content of factors of the BIS-11 are unstable and recommended that researchers explore the items rather than apply the original model [24]. Within Singapore, a multi-ethnic nation-state in Southeast Asia, the BIS-11 has been used in adolescent [25, 26] and pathological gambling [27] samples. However, to date, no studies have utilized a validated measure of impulsivity in Singapore. Therefore, the study aims were twofold: i) to validate the factor structure of the BIS-11 within a sample of outpatients with schizophrenia or other psychotic disorders, and ii) to examine sociodemographic and clinical correlates, (i.e., problematic alcohol use, hazardous drug use, and symptom severity) of impulsivity.

## Method

### Setting and data collection

Data were obtained via a cross-sectional survey conducted at the Institute of Mental Health, a tertiary psychiatric hospital in Singapore. The data for this study were collected as part of a larger study that examined aggression among patients with schizophrenia and psychotic disorders. Hence, sample size calculation was based on the prevalence of 37% of aggression among patients with first-episode psychosis in Singapore [28]. In all, 358 subjects were needed to achieve a 5% precision of estimation at the 95% confidence interval level. Assuming that 10% of patients may not complete the questionnaires or withdraw from the study, a sample size of 400 patients was considered to be sufficient [29].

All respondents were seeking treatment at outpatient clinics and were recruited via convenience sampling. Emails were sent to treating clinicians and other health care professionals requesting them to refer eligible patients. Study brochures containing information on the eligibility criteria and contact details of study team members were displayed in the outpatient clinics. Inclusion criteria for the study were: i) having a clinical diagnosis of schizophrenia and other psychotic disorders (schizoaffective disorder, brief psychotic disorder, delusional disorder, psychosis NOS, and schizophreniform disorder) as determined by a psychiatrist (using Diagnostic and Statistical Manual of Mental Disorders, fourth edition [30] criteria); ii) Singapore citizens or permanent residents; iii) aged between 21 and 65 years, iii) able to understand and read English. Participants who had intellectual disabilities, cognitive impairments, were illiterate in English and were incapable of providing consent were excluded from the study. The study commenced in October 2019 but was suspended during the lockdown period (April 2020 – June 2020) in Singapore due to the Coronavirus pandemic. It was resumed in late June 2020 while adhering to safe distancing and masking policies and providing participants with the option to participate via an online questionnaire. Recruitment was closed at the end of March 2021 once an adequate sample size was achieved. Data were thus collected through both self-administered physical or online questionnaires in the English language. In all, 378 participants completed the study procedures face-to-face, and 19 participants completed it online/electronically**.**

All study procedures were conducted in accordance with ethical guidelines, and ethics approval was received from the relevant institutional ethics review board (National Healthcare Group Domain Specific Review Board). Written or electronic informed consent was obtained from all respondents before data collection. A sample size of 400 respondents was achieved. However, three cases were excluded: i) recruiting the same person twice, ii) one requested to withdraw from the study, and iii) one was above the age limit of 65 years. Hence, a final sample of 397 respondents was utilized for analysis.

### Measures

#### Barratt’s Impulsivity Scale-11 (BIS-11) [19]

The BIS-11 is the 11^th^ revision of the Barratt Impulsiveness Scale and is a 30-item self-report questionnaire to assess the multifaceted personality/behavioural construct of impulsiveness. It is rated on a four-point Likert scale of 1 = Rarely/Never to 4 = Almost Always/Always. The total scores can range from 30 to 120. Higher scores on the BIS reflect higher levels of impulsiveness. Within a sample of undergraduates, psychiatric patients, and inmates, Patton et al. suggested that the BIS-11 comprised six first-order factors of attention, motor impulsiveness, self-control, cognitive complexity, perseverance, and cognitive instability [19]. These fell under three second-order factors: attentional, motor, and non-planning impulsiveness. Examples of items from the scale (each of the subscales) are—‘I make up my mind quickly’ (motor impulsiveness), ‘I often have extraneous thoughts when thinking (attentional impulsiveness), and ‘I say things without thinking’ (non-planning impulsiveness). The BIS-11 has shown good internal consistency in various populations, with Cronbach’s alpha ranging from 0.69 to 0.83 [24, 31].

#### Symptoms Checklist-90-Revised (SCL-90-R) [32]

The SCL-90-R is a 90-item self-report questionnaire that evaluates distress and severity of general psychopathology. Each item is rated on a five-point Likert scale of 0 = Not at all to 4 = Extremely, based on how much the individual was bothered by a particular symptom within the last week. A global severity index (GSI) can be obtained by taking the average of all 90-items, with higher scores reflecting higher distress and severity of symptoms. Psychometric studies for the SCL-90-R have yielded support for the unidimensional score of the scale [33, 34]. It is one of the most widely used measures of psychological distress in clinical practice and research. The questionnaire was included to assess the association of impulsivity with symptoms severity as suggested by others [35, 36]. The internal consistency of the scale was high in the present sample at 0.99.

#### Cut-Annoyed-Guilty-Eye (CAGE) questionnaire [37]

The CAGE questionnaire is a four-item screening tool used to assess lifetime self-reported problems related to alcohol use. It is answered on a dichotomous response scale of 0 = no and 1 = yes. The four items assess “Cutting down,” “Annoyance by criticism,” “Guilty feeling,” and “Eye-opener” related to alcoholism. These four items were prefaced with a screening question of “Was there ever a period in your life when you drank at least 12 drinks in a year?”. Those who had indicated that they never consumed alcoholic drinks or drank less than 12 drinks per year were directed to skip the four CAGE questions, and were classified as having “no drinking problems.” Respondents who had scored two or greater (i.e., endorsing at least two of the four items of the CAGE questionnaire) were classified as having “problematic alcohol use.” The CAGE questionnaire demonstrated moderate to high sensitivity and specificity against a criterion of alcohol abuse and dependence [38] and has been validated in studies amongst older adults in Singapore [39, 40]. Alcohol use disorder has been associated with high levels of impulsivity [41, 42]. CAGE was used in the current study to assess problematic alcohol use in the sample and its association with BIS-11. Internal consistency of the four CAGE items in the present study was moderate, at 0.69.

#### Drug Abuse Screening Test (DAST-10) [43]

The DAST-10 is a 10-item self-report measure that assesses hazardous drug use within the past year. Responses to items about drug use behaviours (e.g., “Have you neglected your family because of your use of drugs?”) are measured on a dichotomous scale of 0 = no and 1 = yes. Responses to each item are summed to provide a total count of hazardous drug use behaviours. Research has demonstrated that a cut-off of ≥ 3 had high sensitivity and specificity against a criterion of drug use disorder [44, 45]. Therefore this cut-off was applied to identify individuals with hazardous drug use. Internal consistency of the DAST-10 items within the present sample was high at 0.73. The DAST-10 was included to assess the association of impulsivity with substance use disorders as suggested by previous research [46].

A structured questionnaire was used to collect sociodemographic information such as age, gender, ethnicity, education, marital status, and monthly personal income (in Singapore dollars (SGD)).

### Statistical analysis

Analyses in the present study were conducted with RStudio 4.0.3. Exploratory Factor Analyses (EFA) were performed using the “psych” package, while Confirmatory Factor Analyses (CFA) were conducted with the “lavaan” package. CFAs were first conducted to test factor structures of the BIS-11 proposed by Patton et al. [19], Ireland and Archer [23], Haden and Shiva [22], Spinella [47], Coutlee et al. [21], and Ros et al. [48]. As items of the BIS are measured on an ordinal four-point scale, a weighted least squares with mean- and variance-adjusted (WLSMV) estimator was used to model the underlying polychoric correlation matrix with pairwise missing data. The following fit indices were utilized to compare the overall fit of the models and their complexities: i) root mean square error of approximation (RMSEA), ii) comparative fit index (CFI), iii) Tucker-Lewis index (TLI), iv) Standardized Root Mean Square Residual (SRMR). Both CFI and TLI values range from 0 to 1, with higher values representing better fit. CFI values above 0.95 and TLI values above 0.90 are considered to be of excellent fit [49]. With regard to RMSEA, values below 0.08 indicate moderate fit [50]. Standardized root mean squared residual (SRMR) values were also evaluated, which indicates acceptable fit when values are smaller than 0.08 [49, 50].

As the factor structures proposed in extant literature demonstrated poor fit to the observed data, the present study adopted a split-half approach, in which the sample (*n* = 397) was randomly divided into two. EFAs were conducted within the first subsample (*n* = 200) to extract the underlying factors. The polychoric correlation matrix with pairwise deletion of missing data was examined with weighted least squares (WLS) estimator and employed an oblique (PROMAX) rotation to obtain a more discriminating factor structure. The following criteria were utilized to determine the number of factors in the EFA: i) eigenvalues > 1 ii) visual inspection of scree plot, iii) results of a parallel analysis, iv) identification of factor loadings on each factor (i.e., loadings > 0.3, cross loadings), and v) the robustness of interpretability for each solution. During each analysis, the factor loading of the items were explored and each rotated solution was examined to identify and remove items that had loadings of < 0.3 and/or cross loadings. The final solution derived from the EFA was then modelled within the second subsample (*n* = 197) for CFA. Subsequently, three linear regressions were conducted to examine associations between sociodemographic variables (i.e., age, gender, ethnicity, education, marital status, and monthly personal income), clinical variables (i.e., hazardous drug use, problematic alcohol use, and symptom severity) and factors derived from the final CFA model.

## Results

### Sociodemographic and clinical characteristics of the sample

Sociodemographic and clinical characteristics of the sample (*n* = 397) are presented in Table 1. Approximately half (49.87%, *n* = 198) were aged 21 to 34, and male (50.63%, *n* = 201). Most of the sample were of Chinese ethnicity (74.81%, *n* = 297) and were single (80.60%, *n* = 320). Of the sample, 5.29% (*n* = 21) reported problematic alcohol use, and 5.79% (*n* = 23) had hazardous drug use.

**Table 1:** Sociodemographic and clinical characteristics of the sample (*N* = 397)

Categorical variables				n			%	
**Age group**								
21–34				198			49.87%	
35–49				146			36.78%	
50–64				53			13.35%	
**Gender**								
Male				201			50.63%	
Female				196			49.37%	
**Ethnicity**								
Chinese				297			74.81%	
Malay				51			12.85%	
Indian				38			9.57%	
Others				11			2.77%	
**Highest education level**								
Primary and below				18			4.53%	
Secondary School				120			30.23%	
Pre-U/Junior College/ Vocational Institute/ITE/Diploma				182			45.84%	
Degree, professional certification, and above				77			19.40%	
**Marital status**								
Single				320			80.60%	
Married				47			11.84%	
Divorced^b^				26			6.55%	
Separated^b^				3			0.76%	
Widowed^b^				1			0.25%	
**Monthly Personal Income (in Singapore Dollars)**								
No income				114			28.72%	
Below 2,000				202			50.88%	
2,000 to 3,999				48			12.09%	
4,000 & above				15			3.78%	
Refused / Don’t Know^a^				18			4.53%	
**Lifetime alcohol problems (CAGE)**								
No lifetime drinking problem (< 2)				376			94.71%	
Problematic alcohol use (≥ 2)				21			5.29%	
**Drug use (DAST-10)**								
No problematic drug use (< 3)				371			93.45%	
Hazardous drug use (≥ 3)				23			5.79%	
Refused/Don’t Know^a^				3			0.76%	
Continuous variables	n	Mean	S.D		P25	P50		P75
Symptoms Checklist 90 Revised—Global Symptom Index	368	0.94	0.87		0.26	0.67		1.39
BIS – Non-planning impulsivity	394	11.97	3.48		10	12		14.75
BIS – Motor impulsiveness	386	13.03	11.46		9	11		13
BIS – Lack of self-control	390	9.92	3.3		8	9		11

^a^Refused/Don’t know responses were treated as missing data

^b^The divorced, separated, and widowed groups were subsumed into a single group for subsequent regression analyses

### Factor extraction and validation

Descriptive information regarding the 30 items of the BIS-11 can be found in Supplementary Table 1. Fit statistics of CFAs employing factor structures suggested by extant literature and the final model can be found in Table 2. As all proposed solutions demonstrated poor fit, EFAs were conducted in the first subsample. The plot of eigenvalues for the polychoric correlation matrix for the initial 30-items indicated that up to seven-factor solutions were plausible. However, upon examination of each rotated solution, 14 items were removed due to cross-loadings and/or loadings < 0.30. Eigenvalues for the remaining 16 items indicated that one to four factors solutions were viable. A three-factor solution was found to be optimal. Subsequently, this solution was applied in the second subsample via CFA, with results indicating acceptable fit of the model to the observed data (WLSMV χ^2^ (101) = 161.26, RMSEA = 0.06, CFI = 0.97, TLI = 0.96, SRMR = 0.07). Standardized factor loadings of the final three-factor solution can be found in Table 3. No modification indices were specified. Standardized factor loadings of the items to their respective factors ranged from 0.43 to 0.86. The three factors were named: i) Non-planning impulsivity (5-items), ii) Motor impulsiveness (6-items), and iii) Lack of self-control (5-items). Internal consistency assessed by Cronbach alpha values were high, at 0.94, 0.84 and 0.85, respectively. To calculate each factor score, items corresponding to their respective factors were summed. However, the scores on the items belonging to the Non-planning factor were reversed before summation as they were positively worded. Therefore, higher scores on all factors indicate higher impulsivity.

**Table 2:** Fit statistics of tested factor structures

Model by:	Factor structure	Number of items	WLSMV χ2 (df)	RMSEA	CFI	TLI	SRMR
Patton et al. (1995) [19]	Unidimensional	30	χ^2^ (405) = 3109.32, p < 0.001	0.13	0.54	0.51	0.16
Patton et al. (1995) [19]	Six first order factors	30	Model does not converge due to covariance matrix of latent variables being not positive definite				
Patton et al. (1995) [19]	Three second order, six first order	30	χ^2^ (396) = 2972.48, *p* < 0.001	0.13	0.57	0.52	0.16
Ireland and Archer (2008) [23]	Three first order	28	Model does not converge				
Haden and Shiva (2008) [22]	Two first order	24	χ^2^ (251) = 1176.32, *p* < 0.001	0.10	0.83	0.81	0.10
Spinella (2007) [47]	Three factor first order (BIS-15)	15	χ^2^(87) = 652.97, *p* < 0.001	0.13	0.82	0.78	0.10
Coutlee et al. (2014) [21]	Three factor first order	13	χ^2^ (62) = 581.49, *p* < 0.001	0.15	0.85	0.81	0.10
Ros et al. (Ros et al., 2020) [48]	Two factor first order	8	χ^2^ (19) = 106.40, *p* < 0.001	0.11	0.91	0.86	0.07
Final model of the present study in the split half sample	Three factor first order	16	χ^2^ (101) = 161.26, *p* < 0.001	0.06	0.97	0.96	0.07

**Table 3:** Standardized factor loadings and fit indices of final CFA model in second subsample (*n* = 197)

Item number	Item description and their corresponding factors	Standardized Factor Loadings
	**Non-planning impulsivity** (6 items; α = 0.81)	
1	I plan tasks carefully^b^	0.67
7	I plan trips well ahead of time^b^	0.67
12	I am a careful thinker^b^	0.82
13	I plan for job security^b^	0.61
20	I am a steady thinker^b^	0.74
	**Motor Impulsiveness** (6 items; α = 0.79)	
2	I do things without thinking	0.64
11	I squirm at plays or lectures	0.63
14	I say things without thinking	0.68
17	I act on impulse	0.82
18	I get easily bored when solving thought problems	0.63
19	I act on the spur of the moment	0.81
	**Lack of self-control** (5 items; α = 0.71)	
22	I buy things on impulse	0.86
24	I change hobbies	0.50
25	I spend or charge more than I earn	0.76
26	I often have extraneous thoughts when thinking	0.66
27	I am more interested in the present than the future	0.43
**Correlations between latent factors**		
Non-planning impulsivity with Motor impulsiveness		-0.14^a^
Non-planning impulsivity with Lack of self-control		0.12^a^
Motor impulsiveness with Lack of self-control		0.80

All standardized latent factor loadings were statistically significant at *p* < 0.001 unless indicated otherwise

^a^The two indicated factor correlations were not statistically significant

^b^Items on factor A were reversed and summed so that higher scores indicate higher impulsivity

### Sociodemographic and clinical correlates of lack of self-control, motor impulsiveness and non-planning impulsivity

Results of the regression models can be found in Table 4. Education was a significant correlate of Non-planning impulsivity and Lack of self-control. Those with education levels of Primary and below (B: 2.49, *p* = 0.02), Secondary (B:1.48, *p* = 0.01), and pre-university/junior college/vocational institute/polytechnic (B:1.16, *p* = 0.03), showed higher impulsivity in the non-planning domain than those with degrees and above. Those with Primary and below education had higher lack of self-control scores than those with degrees and above (B: 1.76, *p* = 0.046). Participants aged 35 to 49 (B: -1.17, *p* = 0.004) and those aged 50 to 65 (B: -1.27, *p* = 0.03) were associated with lower motor impulsiveness when compared to those aged 21 to 34. Those of Malay ethnicity (B: -1.24, *p* = 0.03), were associated with lower motor impulsiveness than those of Chinese ethnicity, while individuals who were separated/divorced/widowed were associated with higher motor impulsiveness (B: 1.47, *p* = 0.046). Problematic alcohol use were associated with greater lack of self-control (B: 1.93, *p* = 0.01). Hazardous drug use was not significantly associated with all three factors. Higher scores on the SCL-90-R Global Symptom Index (i.e., more severe symptoms) were associated with higher motor impulsiveness (B: 2.26, *p* < 0.001), and greater lack of self-control (B: 1.97, *p* < 0.001).

**Table 4:** Results of multivariable linear regression analyses examining correlates of impulsivity

	Non-planning^a^				Motor impulsiveness^b^				Lack of Self-control^c^			
	B	95% CI		*p*	B	95% CI		*p*	B	95% CI		*p*
		Lower	Upper			Lower	Upper			Lower	Upper	
Age												
21 to 34	ref				ref				ref			
35 to 49	0.13	-0.71	0.97	0.76	**-1.17**	-1.98	-0.37	**0.004**	-0.11	-0.80	0.57	0.74
50 to 65	-0.54	-1.77	0.68	0.38	**-1.27**	-2.45	-0.10	**0.03**	0.15	-0.86	1.16	0.77
Gender												
Male	ref				ref				ref			
Female	0.29	-0.48	1.07	0.45	-0.55	-1.29	0.19	0.14	-0.25	-0.88	0.39	0.74
Ethnicity												
Chinese	ref				ref				ref			
Malay	-0.73	-1.94	0.49	0.24	-**1.24**	-2.39	-0.09	**0.03**	-0.79	-1.78	0.20	0.12
Indian	-0.33	-1.63	0.97	0.62	-0.30	-1.55	0.95	0.63	0.08	-0.98	1.14	0.88
Others	-0.33	-2.72	2.06	0.78	-0.70	-3.07	1.67	0.56	-0.52	-2.48	1.44	0.60
Highest education level												
Degree, professional certification, and above	ref				ref				ref			
Primary and below	**2.49**	0.38	4.60	**0.02**	0.27	-1.81	2.34	0.80	**1.76**	0.03	3.50	**0.046**
Secondary School	**1.48**	0.32	2.64	**0.01**	0.14	-0.96	1.25	0.80	0.29	-0.67	1.24	0.56
Pre-U/Junior College Vocational Institute/ITE/Diploma	**1.16**	0.13	2.19	**0.03**	0.05	-0.93	1.04	0.91	0.13	-0.72	0.99	0.76
Marital Status												
Single	ref				Ref				ref			
Married	-0.31	-1.51	0.89	0.61	0.74	-0.41	1.90	0.21	0.20	-0.78	1.19	0.68
Divorced/Separated/Widowed	-0.27	-1.80	1.26	0.72	**1.47**	0.02	2.92	**0.046**	0.45	-0.79	1.69	0.47
Monthly Personal Income (SGD)												
No income	ref				ref				ref			
Below $2,000	-0.27	-1.13	0.60	0.54	0.31	-0.52	1.13	0.47	0.49	-0.22	1.20	0.18
$2,000—$3,999	-0.66	-1.96	0.65	0.32	-0.09	-1.33	1.14	0.88	0.42	-0.65	1.48	0.44
$4,000 and above	-0.13	-2.23	1.96	0.90	0.42	-1.57	2.41	0.68	-0.28	-2.00	1.44	0.75
Lifetime alcohol problems (CAGE)												
No lifetime drinking problem	ref				ref				ref			
Problematic alcohol use	1.29	-0.47	3.05	0.15	1.37	-0.32	3.06	0.11	**1.93**	0.49	3.38	**0.01**
Drug abuse (DAST-10)												
No hazardous drug use	ref				ref				ref			
Hazardous drug use	0.89	-0.81	2.59	0.30	0.24	-1.37	1.86	0.77	-0.43	-1.86	1.01	0.56
Global Symptom Index (SCL-90R)	0.43	-0.02	0.88	0.06	**2.26**	1.83	2.69	** < 0.001**	**1.97**	1.60	2.34	** < 0.001**

*B* Unstandardized Regression Coefficient, *95% CI* 95% confidence interval of B, *SGD* Singapore dollars

Bold print highlights statistically significant B

^a^After accounting for listwise deletion of missing data, cases in linear regression model: 348

^b^After accounting for listwise deletion of missing data, cases in linear regression model: 343

^c^After accounting for listwise deletion of missing data, cases in linear regression model: 347

## Discussion

### Factor structure of the BIS-11

The current study is the only research in Singapore that has examined the factor structure of the BIS-11. The factor models proposed within extant literature were of poor fit within this population and should not be utilized. As recommended by a systematic review by Vasconcelos et al. [24], the present study opted to examine the factor structure of the BIS-11 rather than apply its original model. The results of our study indicate that instead of the original 30-item version, an abbreviated 16-item version of BIS-11 comprising three factors shows better psychometric properties in the Singapore patient sample. Few studies have validated the original factor structure of BIS-11 [51, 52]. The differences may be due to the patient population, as few studies have validated the factor structure among patients with schizophrenia and other psychotic disorders. The three factors identified in our study, non-planning impulsivity, motor impulsiveness, and lack of self-control, reflect the findings in other studies that show that the factor of “non-planning impulsiveness” tends to be the most stable factor, whereas “attentional impulsiveness” is unstable with the items loading on other factors [24]. It is also plausible that cultural and linguistic interpretations of the items of the BIS-11 may have affected its factor structure. For example, the item “I change residences” may not be applicable in Singapore, which according to the commercial real estate service and investment firm CBRE Group, has the 2^nd^ most expensive housing market in the world [53], thus leading to fairly stable living arrangements.

### Education and impulsivity

We found that lower education was associated with higher non-planning impulsivity and a greater lack of self-control. De Wit et al. found that education was negatively related to delayed discounting (a behavioural measure of impulsivity) and scores on the BIS [54]. The authors suggested that individuals with fewer years of education may choose smaller immediate rewards over larger rewards. Similarly, a systematic review by Reimers et al. on the delayed discounting task found that participants who had less education were more likely to choose a smaller-sooner sum of money [55]. This can be corroborated by items in the non-planning factor, which appear to deal with long-term planning.

In contrast, items on the ‘lack of self-control’ deal with immediate rewards. There is a dearth of literature examining how education is related to impulsivity amongst psychiatric samples. However, in a sample of participants with bipolar disorder, education was inversely correlated with the attention and non-planning impulsivity subscales, but not the motor impulsiveness subscale of the BIS-11 [56]. It is unclear why education was not significantly associated with motor impulsiveness, but future studies could examine this in further detail.

### Age, ethnicity, marital status, and motor impulsiveness

Those of Malay ethnicity were found to have higher motor impulsiveness. Studies in the United States have highlighted ethnic differences in impulsivity and suggested that these differences are likely due to environmental factors such as lower socioeconomic status [57–59]. However, the effect of income was adjusted for in the regression models. It was also not a significant correlate in the present results, suggesting factors other than socioeconomic status in play. For example, the large majority of Malays in Singapore endorse Islamic religious beliefs, and research has indicated that religious involvement is inversely related to impulsivity [60]. Similarly, a longitudinal study by Bartkowski et al. has shown that religious families tended to have less impulsive/overactive children [61]. It is plausible that religiosity is a confounding variable related to the motor impulsivity factor. Nevertheless, this ethnic difference in impulsivity should be explored further in future studies.

Marital status has been shown to be related to impulsivity in extant literature. For example, impulsivity in one partner is inversely associated with relationship stability and satisfaction of both partners [62, 63]. Derrick et al. posited that impulsivity affects relationship satisfaction due to the greater and more frequent negative behaviour, fewer pro-social behaviours, and lower likelihood of being responsive to their partner’s needs [64]. Results of the present study indicated that the divorced/separated/widowed group had higher motor impulsiveness than those who were single. Post-hoc pairwise comparisons indicated that the married group did not significantly differ from the single or the divorced/separated/widowed group. It is plausible that those with high impulsivity were at higher risk of a marriage breaking down due to their impulsive behaviour, which may have been associated with aggression or poor functioning [7].

Age differences in impulsivity as identified in the current study, have been well highlighted in existing research. Within a sample of individuals aged 10 to 30 years, Steinberg et al. demonstrated a linear decline of impulsivity with increasing age [65]. Similarly, in a sample of adults aged 18 to 45 years, Herman et al. found that age was negatively correlated with behavioural measures of impulsivity [66]. Furthermore, other studies of adolescence to early adulthood report negative correlations between chronological age and impulsivity [67, 68]. However, we acknowledge that the items measuring impulsivity may not have the same meaning among those belonging to the older age group compared to younger age groups. While the BIS-11 items are generally neutral statements, for example, ‘I get easily bored when solving thought problems’, ‘I act on the spur of the moment’ which should carry the same meanings across different age groups; regardless, some statements could have an age-sensitive interpretation., For example, responses are likely to be confounded by health or physical restrictions that come with aging. A recent study by Tsatali et al. found that both factor structure and factor loadings of the BIS differed across younger and older adult populations [69]. The authors suggested that these differences could be due to differences in the construct of impulsivity due to changes in personality across the lifespan.

### Substance use, psychiatric symptoms, and impulsivity

The present study found that problematic alcohol use was associated with a greater lack of self-control. This is supported by research indicating that patients with schizophrenia who have alcohol abuse/dependence had higher impulsivity [15, 70]. It is plausible that items on the ‘lack of self-control’ factor have more to do with immediate rewards, such as “I buy things on impulse” and “I spend or charge more than I earn.” In line with this, behavioural economics studies have suggested that individuals who misuse alcohol have a steeper devaluation of delayed rewards (i.e., a preference for smaller immediate rewards than larger delayed rewards) [71]. This view suggests that this group of individuals prefer smaller but immediate rewards associated with alcohol use (e.g., intoxication and stress-reduction) when alcohol is immediately available than delayed rewards associated with sobriety or moderation (e.g., employment, health, relationships) [71]. This finding suggests a high-risk group that may require interventional support.

Although numerous studies have suggested a link between drug use and impulsivity within the community and psychiatric populations [10, 70], it is interesting to note these variables were not significantly associated with one another in the present sample. This may be due to two reasons. First, Singapore adopts strict measures towards drug use, with penalties for controlled drugs ranging from hefty fines to corporal and/or capital punishment [72]. As such, drug use in this sample may have been underreported for fear of these consequences, even though participants were assured that their responses were anonymous and confidential. Second, there is a lack of access to drugs within Singapore due to rigorous drug enforcement. It is possible that individuals with high impulsivity might want to use drugs but were unable to do so due to the strict enforcement in place.

Symptom severity as assessed by the SCL-90-R GSI was associated with greater motor impulsiveness and lack of self-control in the current study. Similarly, a recent study within the Hungarian population demonstrated that higher psychological symptoms were correlated with higher impulsivity [36]. Moreover, studies among patients with schizophrenia and other psychotic disorders also suggest that impulsivity is related to general psychopathology, positive and/or negative symptoms [4, 8, 13]. Several mechanisms have been postulated to explain this association. These include the association of severe positive symptoms with disturbances in inhibitory control functions [35, 73] and white matter tracts in the right prefrontal cortex and the right frontal pole that may underlie both dysfunctional impulse control and symptom severity [74].

### Limitations and avenues for future research

The present study contains some limitations that may limit the validity of the findings. Firstly, as the present study is of a cross-sectional nature, it is limited in its ability to identify causal relationships. Secondly, since participants were recruited via convenience sampling, selection bias may have affected the results. We acknowledge that the majority of the sample was young, single, and of Chinese ethnicity. However, the Chinese are the largest ethnic group in Singapore, comprising three-fourths of the population [75], which is also reflected in our patient population. Patients with schizophrenia and other psychotic disorders seen in the clinical settings are generally single and belong to the younger age groups, as seen in several studies which have been conducted in the same population [76, 77]. Thus, the participants were largely representative of the clinical population. However, it is possible that the sample consisted of individuals who were less symptomatic or had less severe symptoms. Given the requirements of the informed consent and self-report, those with severe impairment were unable to participate in this study. This could have affected the results limiting the generalizability of the findings. Thirdly, the data collection period overlapped with the COVID-19 pandemic, and patients may have been more anxious and isolated, thus manifesting a lower level of functioning during this pandemic. This might have influenced participation and further increased the risk of selection bias. Fourthly, the study did not recruit those above 65 years, and the proportion of patients in the 50 -64 age group was relatively smaller. Lastly, it would have been prudent to compare this sample with healthy controls for a more complete picture. Given some of the limitations associated with the sampling, our study may not sufficiently represent the population of those experiencing psychotic disorders.

The present study offers several avenues for future research. First, psychometric properties such as convergent and divergent validity or measurement invariance of the model should be assessed. Second, future studies should examine how different levels of impulsivity are related to actual risk-taking behaviours. For example, researchers could examine the impulsivity levels and forensic profiles (e.g., drug use, violent behaviour) amongst outpatients, inpatients who engage in risky or violent behaviour prior to admission, or those who engage in these behaviours while admitted. Third, future studies examining impulsivity and its correlates can also employ latent class analyses to identify subclasses that may be more prone to risky behaviour. Identifying such groups would provide further understanding and opportunities to modify risk factors.

## Conclusion

The present study found that factor structures by the developers of the BIS-11 were not applicable in a sample of patients with schizophrenia or other psychotic disorders within Singapore. Future studies should examine its underlying factor structure before conducting any subsequent analyses. Results of EFA and CFA indicated a three-factor solution with 16-items: non-planning impulsivity, motor impulsiveness, and lack of self-control. Age, ethnicity, marital status, education, problematic alcohol use, and general psychiatric symptom severity were found to be correlates of impulsivity. These groups may be at-risk for real-world risky or dangerous behaviours, and future studies should examine this relationship in further detail. Mental health services should employ interventions to help these individuals manage their impulsivity.

## Supplementary Information

**Additional file1:** **Supplementary Table 1.** Frequency count of the initial 30-items of the BIS-11.

## Data Availability

The datasets used and/or analysed during the current study are available from the corresponding author on reasonable request.
