# Supplementary material for: The factor structure of the Barratt Impulsiveness Scale (BIS-11) and correlates of impulsivity among outpatients with schizophrenia and other psychotic disorders in Singapore
Source: BMC Psychiatry. 2022 Mar 31;22:226. doi: 10.1186/s12888-022-03870-x (PMC8968701; doi:10.1186/s12888-022-03870-x)
Supplement: Supplementary file 1 — Additional file1: Supplementary Table 1. Frequency count of the initial 30-items of the BIS-11. [file 12888_2022_3870_MOESM1_ESM.docx]

| Supplementary Table 1 | | | | | | | | | | | |
| --- | --- | --- | --- | --- | --- | --- | --- | --- | --- | --- | --- |
| Frequency count of the initial 30-items of the BIS-11 | | | | | | | | | | | |
|  |  | Rarely / Never | | Occasionally | | Often | | Almost Always / Always | | Missing | |
| # | Description | n | % | n | % | n | % | n | % | n | % |
| 1 | I plan tasks carefully ^R^ | 46 | 11.59 | 119 | 29.97 | 154 | 38.79 | 78 | 19.65 | 0 | 0 |
| 2 | I do things without thinking | 156 | 39.29 | 163 | 41.06 | 49 | 12.34 | 26 | 6.55 | 3 | 0.76 |
| 3 | I make-up my mind quickly* | 57 | 14.36 | 169 | 42.57 | 118 | 29.72 | 51 | 12.85 | 2 | 0.5 |
| 4 | I am happy-go-lucky* | 75 | 18.89 | 131 | 33.00 | 105 | 26.45 | 85 | 21.41 | 1 | 0.25 |
| 5 | I don’t “pay attention”* | 132 | 33.25 | 180 | 45.34 | 52 | 13.10 | 29 | 7.30 | 4 | 1.01 |
| 6 | I have “racing” thoughts* | 140 | 35.26 | 147 | 37.03 | 74 | 18.64 | 36 | 9.07 | 0 | 0 |
| 7 | I plan trips well ahead of time ^R^ | 62 | 15.62 | 126 | 31.74 | 139 | 35.01 | 69 | 17.38 | 1 | 0.25 |
| 8 | I am self-controlled* | 33 | 8.31 | 104 | 26.2 | 167 | 42.07 | 91 | 22.92 | 2 | 0.5 |
| 9 | I concentrate easily* | 38 | 9.57 | 153 | 38.54 | 145 | 36.52 | 58 | 14.61 | 3 | 0.76 |
| 10 | I save regularly* | 65 | 16.37 | 142 | 35.77 | 108 | 27.20 | 77 | 19.40 | 5 | 1.26 |
| 11 | I “squirm” at plays or lectures | 179 | 45.09 | 128 | 32.24 | 50 | 12.59 | 38 | 9.57 | 2 | 0.5 |
| 12 | I am a careful thinker ^R^ | 36 | 9.07 | 115 | 28.97 | 167 | 42.07 | 79 | 19.9 | 0 | 0 |
| 13 | I plan for job security ^R^ | 74 | 18.64 | 114 | 28.72 | 131 | 33.00 | 78 | 19.65 | 0 | 0 |
| 14 | I say things without thinking | 153 | 38.54 | 163 | 41.06 | 53 | 13.35 | 25 | 6.3 | 3 | 0.76 |
| 15 | I like to think about complex problems* | 109 | 27.46 | 134 | 33.75 | 85 | 21.41 | 69 | 17.38 | 0 | 0 |
| 16 | I change jobs* | 128 | 32.24 | 134 | 33.75 | 73 | 18.39 | 55 | 13.85 | 7 | 1.76 |
| 17 | I act “on impulse” | 141 | 35.52 | 156 | 39.29 | 64 | 16.12 | 35 | 8.82 | 1 | 0.25 |
| 18 | I get easily bored when solving thought problems | 138 | 34.76 | 150 | 37.78 | 78 | 19.65 | 28 | 7.05 | 3 | 0.76 |
| 19 | I act on the spur of the moment | 151 | 38.04 | 168 | 42.32 | 51 | 12.85 | 27 | 6.80 | 0 | 0 |
| 20 | I am a steady thinker ^R^ | 54 | 13.60 | 133 | 33.50 | 147 | 37.03 | 61 | 15.37 | 2 | 0.5 |
| 21 | I change residences* | 272 | 68.51 | 90 | 22.67 | 23 | 5.79 | 10 | 2.52 | 2 | 0.5 |
| 22 | I buy things on impulse | 154 | 38.79 | 147 | 37.03 | 56 | 14.11 | 39 | 9.82 | 1 | 0.25 |
| 23 | I can only think about one thing at a time* | 84 | 21.16 | 136 | 34.26 | 108 | 27.2 | 69 | 17.38 | 0 | 0 |
| 24 | I change hobbies | 233 | 58.69 | 106 | 26.7 | 37 | 9.32 | 21 | 5.29 | 0 | 0 |
| 25 | I spend or charge more than I earn | 169 | 42.57 | 129 | 32.49 | 57 | 14.36 | 38 | 9.57 | 4 | 1.01 |
| 26 | I often have extraneous thoughts when thinking | 123 | 30.98 | 149 | 37.53 | 72 | 18.14 | 52 | 13.1 | 1 | 0.25 |
| 27 | I am more interested in the present than the future | 87 | 21.91 | 154 | 38.79 | 93 | 23.43 | 62 | 15.62 | 1 | 0.25 |
| 28 | I am restless at the theatre or lectures* | 163 | 41.06 | 127 | 31.99 | 64 | 16.12 | 43 | 10.83 | 0 | 0 |
| 29 | I like puzzles* | 154 | 38.79 | 118 | 29.72 | 76 | 19.14 | 49 | 12.34 | 0 | 0 |
| 30 | I am future oriented* | 71 | 17.88 | 128 | 32.24 | 124 | 31.23 | 74 | 18.64 | 0 | 0 |
| * indicates items that were removed from the final EFA/CFA models  ^R^ indicates items that were reversed scored | | | | | | | | | | | |
